# AKT Signaling Mediates IGF-I Survival Actions on Otic Neural Progenitors

**DOI:** 10.1371/journal.pone.0030790

**Published:** 2012-01-23

**Authors:** Maria R. Aburto, Marta Magariños, Yolanda Leon, Isabel Varela-Nieto, Hortensia Sanchez-Calderon

**Affiliations:** 1 Instituto de Investigaciones Biomedicas “Alberto Sols”, CSIC-UAM, Madrid, Spain; 2 CIBERER, Unit 761, ISCIII, Madrid, Spain; 3 Departamento de Biologia, Universidad Autonoma de Madrid, Madrid, Spain; Universidade Federal do Rio de Janeiro, Brazil

## Abstract

**Background:**

Otic neurons and sensory cells derive from common progenitors whose transition into mature cells requires the coordination of cell survival, proliferation and differentiation programmes. Neurotrophic support and survival of post-mitotic otic neurons have been intensively studied, but the bases underlying the regulation of programmed cell death in immature proliferative otic neuroblasts remains poorly understood. The protein kinase AKT acts as a node, playing a critical role in controlling cell survival and cell cycle progression. AKT is activated by trophic factors, including insulin-like growth factor I (IGF-I), through the generation of the lipidic second messenger phosphatidylinositol 3-phosphate by phosphatidylinositol 3-kinase (PI3K). Here we have investigated the role of IGF-dependent activation of the PI3K-AKT pathway in maintenance of otic neuroblasts.

**Methodology/Principal Findings:**

By using a combination of organotypic cultures of chicken (*Gallus gallus*) otic vesicles and acoustic-vestibular ganglia, Western blotting, immunohistochemistry and *in situ* hybridization, we show that IGF-I-activation of AKT protects neural progenitors from programmed cell death. IGF-I maintains otic neuroblasts in an undifferentiated and proliferative state, which is characterised by the upregulation of the forkhead box M1 (FoxM1) transcription factor. By contrast, our results indicate that post-mitotic p27^Kip^-positive neurons become IGF-I independent as they extend their neuronal processes. Neurons gradually reduce their expression of the *Igf1r*, while they increase that of the neurotrophin receptor, TrkC.

**Conclusions/Significance:**

Proliferative otic neuroblasts are dependent on the activation of the PI3K-AKT pathway by IGF-I for survival during the otic neuronal progenitor phase of early inner ear development.

## Introduction

The vertebrate inner ear is a complex sensory organ responsible for the senses of sound and balance. The inner ear derives from an ectodermal placode that invaginates to form the otic vesicle, or otocyst. This structure contains the information required to carry out an autonomous program of development resulting in generation of most of the cells that form the adult inner ear. Inner ear development requires the transition of otic progenitors through states of active cell proliferation, cell fate specification, synchronized cell cycle exit and differentiation to generate the distinctly fated cell populations within the highly ordered mosaic of the organ of Corti in mammals and the basilar papilla in birds [1], [2], [3]. Neurons of the acoustic and vestibular ganglia have a common origin in the otic placode. Neuronal progenitors are specified in the neurogenic otic epithelia, from where they migrate to form the acoustic-vestibular ganglion (AVG) [2]. The AVG later develops into the acoustic and vestibular ganglia that connect the sensory epithelia to the brain through the VIII cranial nerve [4]. Programmed cell death contributes to otic development and neurogenesis by controlling neuroblast cell number [5], [6], [7]. Neurodevelopmental programmed cell death occurs either in proliferating progenitors or upon loss of neurotrophins [8]. The postmitotic dependence of otic neurons on neurotrophins has been the subject of intense study [9], [10], [11], but the regulation of cell death during the expansion period of proliferative otic neuroblasts remains poorly understood.

IGF-I deficiency is associated with severe cochlear defects and human sensorineural deafness (http://www.ncbi.nlm.nih.gov/omim/608747), [12], [13], [14], [15]. In the chicken embryo, IGF-I is required for the early morphogenesis and survival of otic vesicle epithelial cells [16], [17], as well as for AVG neurogenesis [18]. Binding of IGF-I to high affinity receptors, IGF1R, activates two main downstream signalling pathways, namely the RAF-MEK-ERK phosphorylation cascade and the phosphatidylinositol 3 kinase-AKT (PI3K-AKT) pathway [7], [19], [20]. *Igf1* ^−/−^ null mouse cochleae showed decreased activation of the PI3K-AKT pathway and an altered FoxM1/p27^kip^ ratio when compared to wild type mice [21]. In addition, the genomic analysis of the null mutant mouse for the zinc finger transcription factor *Gata3*, a fundamental player in auditory system development [22], has shown alterations in the expression of genes linked to IGF-signalling, including AKT and p27^Kip^ [23]. Still, a detailed study of the role of AKT during inner ear development has not yet been performed.

The serine-threonine protein kinase AKT (http://www.ncbi.nlm.nih.gov/omim/164730) is activated by binding of phosphatidylinositol 3-phosphate, which promotes its recruitment to the plasma membrane and sequential phosphorylation at threonine (Thr308) and serine (Ser473) residues by the phosphoinositide dependent kinases [24]. AKT can then phosphorylate and inactivate downstream proteins implicated in programmed cell death [24], [25] and cell cycle regulation, such as the cyclin-dependent kinase inhibitor p27^Kip^ [26], [27], [28]. The study of knockout mice for the *Akt*1 and *Akt*2 genes has shown that they play a fundamental role in cell growth and survival [29], [30], [31]. Furthermore, Akt1 levels regulate survival, proliferation and self-renewal of neural progenitors in response to extrinsic signals [31].

Here we show that AKT activation is fundamental for the IGF-I-dependent survival of the expanding population of otic neuroblasts.

## Materials and Methods

### Chicken embryos

Chicken embryos were obtained from fertilized eggs purchased from a local farm (Granja Santa Isabel, Córdoba, Spain), they were incubated in a humidified atmosphere at 37.8°C and the embryos were staged according to Hamburger and Hamilton criteria (HH; [32]). All experiments were performed following the recommendations of the European Communities Council Directive (86/609/EEC) and they were approved by the Ethics Committee of the CSIC.

### Embryo and tissue preparation for *in situ* hybridization and immunofluorescence

Whole embryos or tissues were dissected in phosphate-buffered saline (PBS) and fixed overnight in 4% paraformaldehyde in PBS at 4°C. Subsequently, embryos were cryoprotected overnight in 15% sucrose/PBS at 4°C and then embedded at 37°C in 15% sucrose/10% gelatine in PBS. Gelatine-embedded tissues were frozen in isopentane at −80°C and then sectioned (20 µm) at −25°C in a cryostat (Cryocut 1900; Leica Microsystems, Deerfield, IL). The sections obtained were used for *in situ* hybridization or immunofluorescent staining.

### *Igfr1* cloning and in situ hybridization

*In situ* hybridization with digoxigenin-labelled antisense RNA probes (1 mg/ml) was performed essentially as described previously with some minor modifications [33]. Three HH19 embryos were tested in parallel in at least two independent experiments and no specific signal was obtained with the control sense probes (data not shown). The ck*Igf1r* gene was cloned by PCR (*Igf1r* forward 5′-CAGTTCTCTCCTTGCCATCC-3′; *Igf1r* reverse 5′-CAGCATCCAACTCCTCTTCC-3′) and ligated into a pGEM-T plasmid (Promega, Madison, WI). The probes for ck*TrkC* were kindly provided by Dr. Martin-Zanca (CSIC-USAL, Salamanca, Spain, [34]). Antisense single-stranded RNA probes for ck*TrkC* (BSU361/T7) and *ckIgf1r* (ApaI/Sp6) were prepared by *in vitro* transcription.

### Ex vivo culture of isolated otic vesicles and AVG

Otic vesicles were dissected from stage HH18 embryos (65 h of incubation), transferred into four-well culture-plates (Nunc, Roskilde, Denmark) and incubated at 37°C in a water-saturated atmosphere containing 5% CO_2_ as described previously [35]. The standard culture medium consisted of M199 medium with Earle's salts (Sigma-Aldrich, Saint Louis, MO) supplemented with 2 mM glutamine (Gibco, Paisley, UK) and antibiotics [50 IU/ml penicillin (Ern, Barcelona, Spain) and 50 mg/ml streptomycin (CEPA, Madrid, Spain)]. AVG were aseptically dissected out from stage HH19 chick embryos (76 h of incubation) [36], they were plated onto glass cover slips previously coated with poly-D-lysine and fibronectin as described in [37]. The AVG was cultured in 0.25 ml F12/Dulbecco's modified Eagle medium (Gibco) containing 100 mg/ml transferrin, 16 mg/ml putrescine, 6 ng/ml progesterone, 5.2 ng/ml sodium selenite (all from Sigma-Aldrich), and antibiotics as above. The cultures were treated for the times indicated in results section as follows: fetal bovine serum (FBS 2.5% v/v), IGF-I (10 nM), PI3K inhibitor LY294002 (LY; 2-(4-Morpholinyl)-8-phenyl-4H-1-benzopyran-4-one; 25 µM), AKT inhibitor VIII (AKTi; 1,3-Dihydro-1-(1-((4-(6-phenyl-1H-imidazo[4,5-g]quinoxalin-7-l)phenyl)methyl)-4-piperidinyl)-2Hbenzimidazol-2-one; 50 µM) or pan-caspase inhibitor (Boc-D-fluoromethyl ketone; Boc-D-FMK; 20 µM). These concentrations were considered optimal since they promoted an effect without producing any toxic reaction, as determined empirically after testing different concentrations of each inhibitor. Recombinant human IGF-I was purchased from Roche Molecular Biochemicals (Basel, Switzerland) [5]. LY294002 was purchased from Cell Signalling (Boston, MA), while Boc-D-FMK and AKTi were purchased from Calbiochem (La Jolla, CA). The solvent used (DMSO) in the culture medium had no detectable effect on explants when used at a final concentration of 0.01% for LY294002, Boc-D-FMK and AKTi cultures. Otic vesicles or AVG cultured in defined medium with no serum were considered as controls (0S). For immunostaining and TUNEL labelling, both otic vesicles and AVG were fixed for 2 h in 4% (w/v) paraformaldehyde (Merck) at 4°C after culture.

### Immunofluorescence

The sources, dilution, and cell specificities of the antibodies used for immunofluorescent staining are shown in Table 1. Samples were washed and permeabilized in 1% or 0.05% PBS/Triton-X-100 (PBS-T) (whole-mount otic vesicles or AVG and frozen sections, respectively). An additional permeabilization step of 30 min in PBS-T at 37°C, was carried out in the explant whole-mount immunostaining. Non-specific binding sites were blocked for 1 h in PBS-T with 3% (wt/vol) BSA (Sigma-Aldrich) and 5% (vol/vol) normal goat or donkey serum. Samples were exposed to the primary antibodies overnight at 4°C, diluted in PBS-Tween20 (0.05%). The fluorescence conjugated secondary antibodies (anti-mouse Alexa488, anti-rabbit Alexa488, anti-mouse Alexa546, anti-rabbit Alexa546, anti-rabbit Alexa647 or anti-goat Alexa660; Molecular Probes, Eugene, OR) were incubated with the samples for 3 h at room temperature (RT) at a dilution of 1∶400 in PBS Tween 20. For dual-fluorescence immunolabelling, samples were incubated with a mixture of fluorescent-conjugated secondary antibodies. The otic vesicles were mounted in Prolong Gold with DAPI (Invitrogen, Carlsbad, CA) and fluorescence staining was visualized by confocal microscopy (Leica TCS SP2, Wetzlar, Germany). At least six otic vesicles per condition were assayed from three independent experiments. For immunohistofluorescent staining of frozen sections, the sections were thawed and the protocol indicated above was followed. The sections were then mounted in Prolong Gold with DAPI (Invitrogen) and they were visualized under a fluorescence microscope (Nikon 90i, Tokyo, Japan).

**Table 1:** Primary antibodies.

Antigen	Type^1^	Source/Cat. No.	Conc.	Technique^2^
Islet-1^3^	MouM	DSHB/39.4D5	1∶3	IHF
		(*supernatant*)		
Tuj-1 (β-III Tubulin)	RbP	Covance (Berkeley,	1∶1000	IHF
		CA)/PRB-435P		
G4 glycoprotein	RbP	[41], [63]	1∶ 500	IHF
SOX2	GtP	Santa Cruz/SC-17320	1∶50	IHF
SOX10	GtP	Santa Cruz/SC-	1∶50	IHF
		17342		
FoxM1	RbP	Santa Cruz/SC-500	1∶500	WB
BrdU	MouM	Sigma-	1∶150	IHF
		Aldrich/B8434		
Active Caspase-3	RbP	Promega/G7481	1∶30	IHF
p27^Kip^	MouM	BD (Franklin Lakes,	1∶50	IHF
		NJ)/610241		
Phospho-Histone-H3	RbP	Upstate/06-570	1∶200	IHF
(PH3)				
AKT	GtP	Santa Cruz/SC-1619	1∶1000	WB
pAKT^Ser 473^	RbP	Cell Signalling/9271	1∶1000	WB/IHF
ERK (p44/42 MAP	RbP	Cell Signalling/9102	1∶1000	WB
kinase)				
pERK (phospho-	RbP	Cell Signalling/9101	1∶1000	WB
p44/42 MAP kinase)				

1 Antibody type: RbP, rabbit polyclonal; MouM, mouse monoclonal; GtP, goat polyclonal.

2 Technique: IHF, Immunohistofluorescence. WB, Western Blotting.

3 Monoclonal antibody developed by Thomas Jessell and Jane Dodd and obtained from the Developmental Studies Hybridoma Bank developed under the auspices of the NICHD, which is maintained by the Department of Biological Sciences, University of Iowa, Iowa City, IA 52242.

Levels of SOX2, G4 and p27^Kip^ immunostaining were quantified using Adobe Photoshop CS4 software (Adobe Systems Inc., CA, USA) in compiled confocal microscopy projections. The colour channels of the signals of interest were converted into grey scale images. Then both the area and the intensity of the signal were measured, and normalised to the 0S condition, which was given an arbitrary value of 100. The data are presented as the mean ± SEM and the statistical significance was estimated with the Student's t-test.

AVG areas (neural processes and neural somas) were measured using the ImageJ software (Wayne Rasband, National Institutes of Health, Bethesda, MD) and the results are presented as the mean ± SEM of the areas. Statistical significance was estimated by Student's t-test.

### Programmed cell death, BrdU incorporation and immunodetection

Cell death was studied by Tdt-mediated dUTP nick-end labelling (TUNEL) staining of fragmented DNA using the kit Dead-End™ Fluorometric TUNEL System (Invitrogen) adapted to whole organ labelling [38]. The otic vesicles were mounted in Prolong Gold/DAPI and visualized by confocal microscopy. To study cell proliferation, otic vesicles were incubated with 5-Bromo-2′-deoxyuridine (BrdU, 10 mg/ml, Sigma-Aldrich) during the last hour of culture. BrdU incorporation was immunodetected as described with additional steps to denature the DNA. Samples were incubated in 50% (vol/vol) formamide-SSC for 40 min at 65°C and in 2N HCl for 30 min at 37°C, followed by a 10 min wash in Tris 0.1 M (pH 8). At least six otic vesicles per condition were assayed from three independent experiments.

TUNEL-positive nuclei were quantified in compiled confocal microscopy projections with Image Analysis Software (Olympus) and the results are presented as the mean ± SEM of the positive cells per total area. Values were normalised to the 0 h time point (otic vesicles fixed immediately after removal from the embryo). Areas from medial sections of the otic vesicle and the AVG were measured using the ImageJ software (Wayne Rasband, National Institutes of Health, Bethesda, MD) and the results are presented as the mean ± SEM of the areas (µm^2^). Statistical significance was estimated by Student's t-test or by ANOVA with Bonferroni and Tukey's multiple comparisons post-hoc tests, using SPSS for Windows 15.0 software (SPSS Inc., Chicago, IL).

### Quantitative RT-PCR

Chicken embryos were dissected out and pooled to obtain RNA at different stages: HH17 (n = 45), HH18 (n = 30), HH19 (n = 25) and HH23 (n = 5). Four independent pools of RNA from each stage were isolated with TRIZOL (Invitrogen) following the manufacturer's instructions. The integrity and concentration of the RNA was assessed with an Agilent Bioanalyzer 2100 (Agilent Technologies) and cDNA was generated by reverse transcription (High Capacity cDNA Reverse Transcription Kit; Applied Biosystems). Real-Time PCR of each pool was performed in triplicate using specific oligonucletides for the available chicken AKT genes (*Akt1* and *Akt3*; Quantitect Primer Assays, Geneglobe, Qiagen). Primers used were: *Akt1* and *Akt3* (QT00593411 and QT01484105, respectively; Geneglobe, Qiagen), using SYBR Green as the detection system. Eukaryotic 18S rRNA was used as the endogenous housekeeping gene (Hs99999901_s1, TaqMan, Applied Biosystems). PCR was performed on a 7900HT Real-Time PCR System and gene expression was estimated as 2^−ΔΔCt^.

### Western blotting

Otic vesicles were made quiescent by incubating overnight in serum-free M199 culture medium; they were pre-treated with the inhibitors (LY and AKTi) for 1 h and then stimulated with IGF-I for 30 min. At the end of this period, the otic vesicles were homogenized in ice cold Laemmli Buffer containing 50 mM dithiotreitol, phosphatase inhibitor cocktail 2 and protease inhibitor cocktail (both 1∶100, from Sigma-Aldrich) and stored immediately at −20°C. Before loading the samples, they were heated at 95°C for 5 min. Electrophoresis, protein transfer and immunodetection were carried out as described [20] using the antibodies shown in Table 1. Four independent experiments were performed using 15 HH18 otic vesicles per condition. Western blot films were scanned and the bands were quantified by densitometry with Adobe Photoshop CS4 software (Adobe Systems Inc.). The results are presented as the mean ± SEM of the phosphorylated AKT (on Ser 473)/AKT ratio. The IGF-I condition was considered as the reference and given a value of 100. The statistical significance was estimated with the Student's t-test.

## Results

### In vivo expression of *Igf1r* in the developing AVG

**Figure 1:**
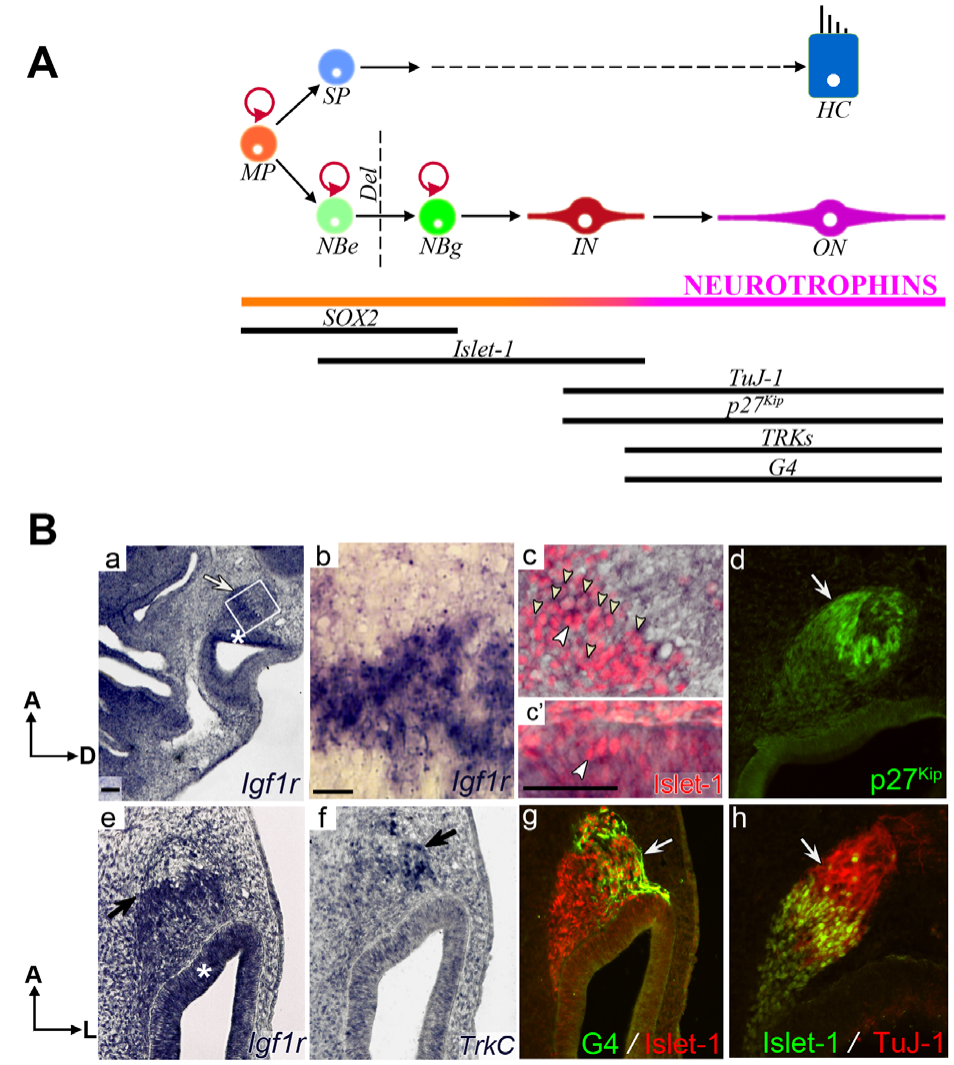
*Igf1r* expression and molecular markers of otic neurogenesis in the developing inner ear. (**A**) Schematic representation of the cellular stages of the development of otic neurons. Abbreviations: Del, delaminating; HC, hair cell; IN, immature neuron; MP, multipotent progenitor; NBe epithelial neuroblast; NBg, ganglionic neuroblast; ON, otic neuron; SP, sensory progenitor. Adapted from [7]. (**B**) *Igf1r* and neural markers expression in the HH19 developing inner ear. *In situ*-hybridization was performed on HH19 chicken embryos for *Igf1r* (a, b, c, c′ and e) and *TrkC* (f) in parasagital (a–d, h) or coronal sections (e–g). The following neural differentiation and post-mitotic markers were studied by immunohistochemistry: Islet-1 (red in c, c′, g; green in h), p27^Kip^ (green in d), G4 (green in g) and TuJ-1 (red in h). *Igf1r* is expressed in the otic epithelium and in the AVG (a and e, asterisks and arrows, respectively)mainly in the ganglionar and epithelial Islet-1-positive neuroblasts (b, c and c′). The arrowheads in c and c′ point to the nuclear Islet-1 expression in the neuroblasts and the smaller arrowheads in c point to the *Igf1r* expression in their cytoplasm. Neurons expressing p27^Kip^ (d) and TuJ-1 (h) coincide spatially with the limit of expression of *Igf1r* (compare b with d and h, arrows). Neurons expressing *TrkC* also express differentiation markers like G4 (f and g, arrows). Panel b corresponds to the boxed area in a; panels c and c′ corresponds to the boxed areas in b. Representative microphotographs are shown from at least three embryos per condition. Orientation: A, anterior; D, dorsal; L, lateral;. Scale bars, 75 µm.

During otic neurogenesis there is a sequence of otic neural maturation, which is reflected by the expression of molecular markers characteristic of the transition from epithelial to ganglionic neuroblasts, then to immature and, finally, to mature neurons (Figure 1A). Islet-1 is a LIM/homeodomain transcriptional regulator that is expressed by early otic neuroblasts [39], [40], and TuJ-1 is a β-III-tubulin that labels fibers in immature ganglionic neuroblasts. The axonal glycoprotein G4 [18], [20] [41] and the TrkC neurotrophin receptor [42] are characteristic of mature otic neurons Neuroblasts are characterized by their capacity to proliferate, whilst neurons are post-mitotic and express cell cycle inhibitors such as p27^Kip^ [43].

To identify the neural otic populations modulated by IGF-I, we studied *Igf1r* expression in the developing HH19 otic vesicle, and its cellular co-localization with neural differentiation markers (Figure 1 B). *Igf1r* was ubiquitously expressed in the developing inner ear at stage HH19, although its expression was notably stronger in the otic epithelium adjacent to the AVG and in the ganglion neuroblasts (Figure 1 B, a and e, asterisks and arrows, respectively). Within the AVG, *Igf1r* expression was intense in neuroblasts strongly labeled for Islet-1 (Figure 1 B, b and c, arrowheads). In the otic epithelium, *Igf1r* and Islet-1 were expressed strongly in the neurogenic zone (Figure 1 B, c′, arrowheads). In striking contrast, the ganglion population most distal to the neurogenic epithelium exhibited a weak *Igf1r* expression, concomitantly with an intense p27^Kip^ label, G4-positive neuronal processes, TuJ-1 and *TrkC* expression (Figure 1 B, d, f, g and h, arrows). These data suggest that undifferentiated otic neuroblasts show high levels of IGF1R, but, as development proceeds, otic neurons down-regulate IGF1R expression.

### Spatial distribution of neural markers in cultured otic vesicle explants

**Figure 2:**
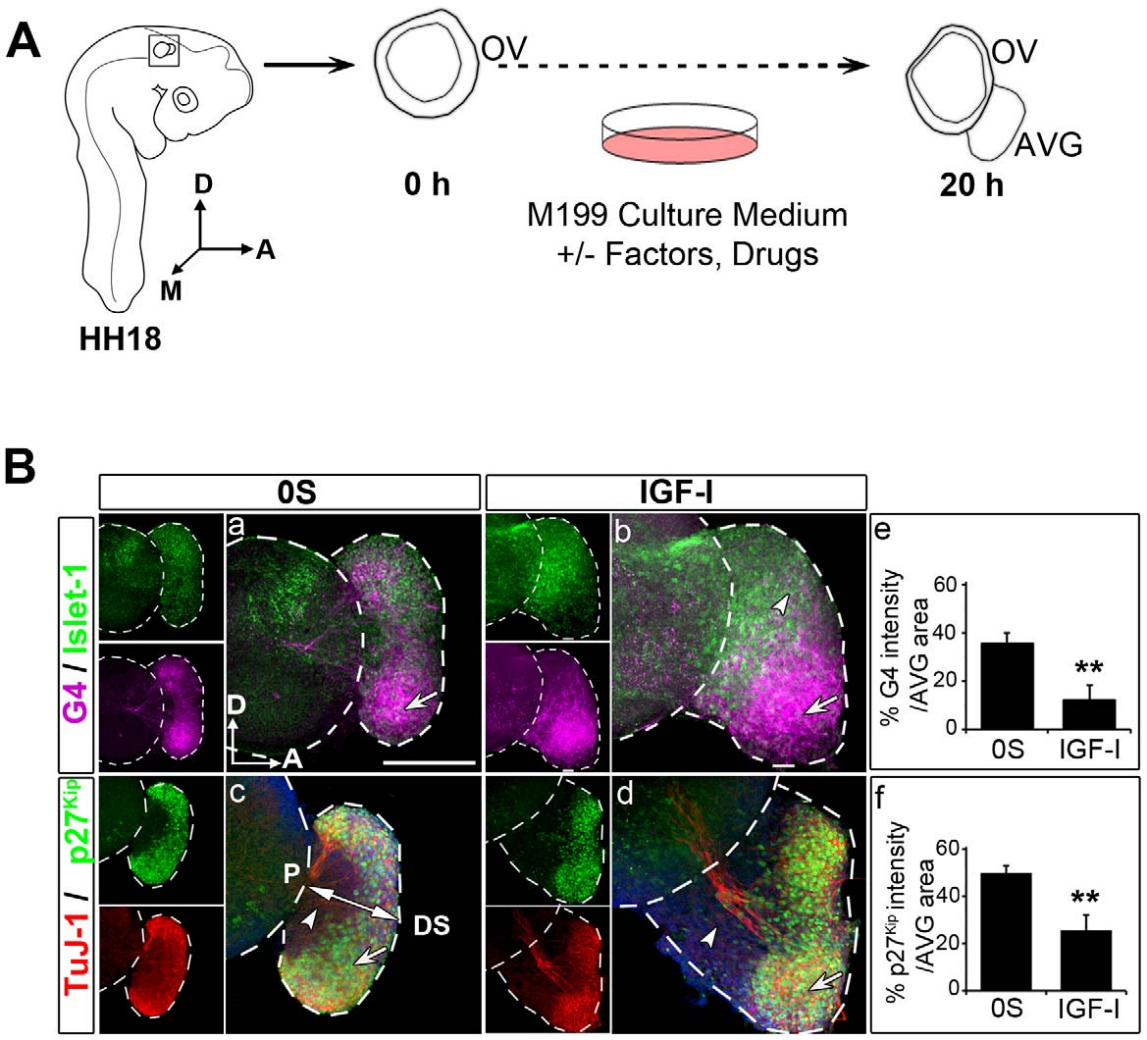
IGF-I modulates the expression of neurogenic markers in cultured otic vesicles. (**A**) Schematic representation of the otic vesicle *ex vivo* culture. The otic vesicle can be explanted from the embryo at HH18. The AVG also develops *ex vivo*, and thus this constitutes an excellent model to study otic neurogenesis. The figure shows a schematic drawing of a HH18 chicken embryo showing the location of the otic vesicle, and of an otic vesicle immediately after dissection (0 h) and after 20 h in culture (20 h). Factors and drugs can be added to the serum-free culture medium to study their effects on the otic vesicle *ex vivo* development and AVG formation. Abbreviations: AVG, acoustic-vestibular ganglion; OV, otic vesicle. (**B**) Neurogenic markers in cultured otic vesicles. Otic vesicles were isolated from HH18 chicken embryos and cultured in serum-free medium without additives (0S; a, c) or supplemented with IGF-I (10 nM; b, d). Upon immunostaining, the spatial expression patterns of neural maturation markers Islet-1 (green in a, b), TuJ-1 (red in c, d) and G4 (magenta in a, b), and of the cyclin-dependent kinase inhibitor, p27^Kip^ (green in c, d) were studied. Arrows indicate the ganglionar populations with the strongest G4 expression (a, b) and TuJ-1 (c, d), and arrowheads show the G4- and p27^Kip^-negative population in the AVG (a–d). The double-headed arrow in c indicates the proximal (P)- distal (DS) axis in the AVG. Representative images of six otic vesicles per condition and from at least three independent experiments are shown, and they were obtained from compiled confocal microscopy projections of otic vesicles. G4 and p27^Kip^ levels were quantified (as described in Materials and Methods) and refereed to the total AVG area to compare their contribution to the AVG size under the different culture conditions. The bars show the mean ± SEM of at least five otic vesicles from any of the conditions shown in a-d. Statistical significance was estimated with the Student's t-test: **P<0.01 versus control (0S). Orientation: A, anterior; D, dorsal; M, medial. Scale bar, 150 µm.

Otic vesicles can be explanted from the embryo and their *ex vivo* development can be followed in a defined culture medium to study the molecular cues that instruct the cellular diversity found *in vivo* [7]. The AVG also develops *ex vivo*, and thus this constitutes an excellent model to study otic neurogenesis [38], [37] (Figure 2 A). To further explore the IGF-I actions in the different neural differentiation stages, we used the *ex vivo* model to compare the neural markers G4, Islet-1, TuJ-1 and the post-mitotic marker p27^Kip^ in the untreated condition (cultured in the absence of any additives; 0S) and in IGF-I-treated otic vesicles (Figure 2 B). In both conditions, the AVG showed areas populated by cells at distinct neural maturation stages. Thus, the most distal and ventral aspect of the AVG showed weaker Islet-1 and stronger TuJ-1/G4 and p27^Kip^ staining than the proximal and dorsal aspects (Figure 2 B, a–d, arrows; c, double-headed arrow). Accordingly, immature G4 and p27^Kip^-negative neuroblasts were confined to the more proximal and dorsal part of the AVG, adjacent to the otic epithelium (Figure 2 B, a–d, arrowheads). Treatment with IGF-I for 20 h resulted in an increased size of the AVG (Figure 2 B, compare a, c with b, d) and the G4-negative cell population was confined to the proximal-dorsal part of the AVG (Figure 2 B, b, arrowhead). Indeed, in the AVG cultured with IGF-I there was a significant reduction in the proportion of G4 positive labeling with respect to the total area when compared with the 0S condition (Figure 2 B, quantification in e). Accordingly, the proportion of postmitotic p27^Kip^- cells was also significantly higher in the IGF-I-treated AVG than in control cultures (Figure 2 B, compare c with d, arrowheads; quantification in f). Thus, IGF-I-treated cultures show a wider p27^Kip^/G4-negative immature neuroblast population than control cultures.

Glial cells for the AVG are derived from the neural crest and can be detected by immunolocalization of the transcription factor SOX10 [37], [44]. In HH19 embryos, the AVG shows SOX10-positive cells associated to Islet-1 positive neuroblasts.In contrast, cultures of otic vesicles develop a ganglion that shows no detectable contribution of glial cells in any of the culture conditions tested, 0S, IGF-I treatment or incubation with LY (Figure S1).

### Spatiotemporal patterns of cell proliferation in cultured otic vesicles

**Figure 3:**
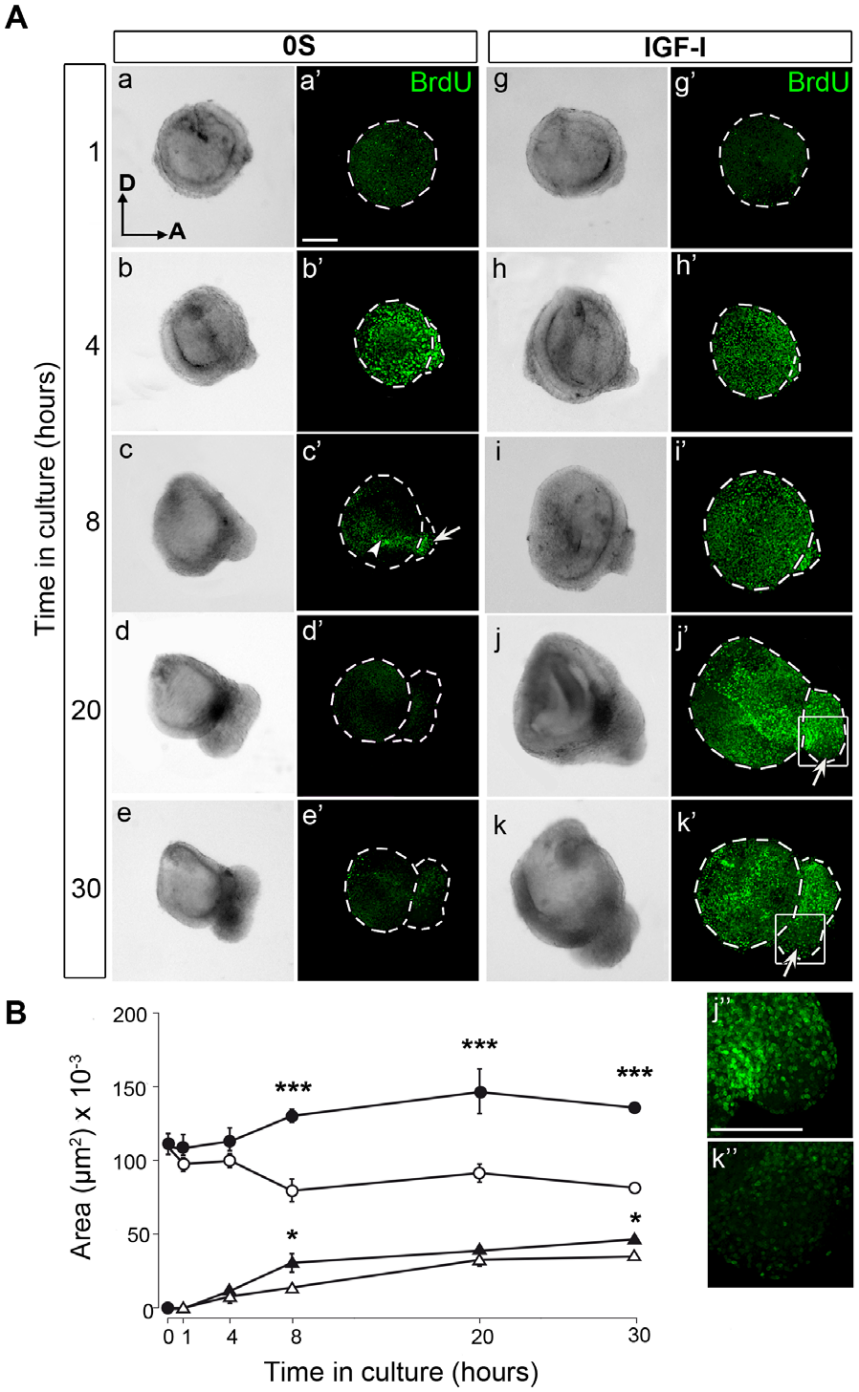
IGF-I promotes BrdU incorporation in the otic vesicle. (**A**) Spatiotemporal pattern of BrdU incorporation in cultured otic vesicles. Otic vesicles were isolated from HH18 chicken embryos and cultured in serum-free medium without additives (0S; a–e and a′–e′) or supplemented with IGF-I (10 nM; g–k and g′–k′). Immunostaining for incorporated BrdU was performed (green), and light microscopy images are shown for comparison (a–k). The arrowhead points to the accumulation of BrdU-positive cells in the neurogenic region in the otic epithelium (c′), whilst the arrows indicate the areas of low BrdU incorporation in the AVG (c′, j′, k′). Panels j″ and k″ correspond to the boxed areas of the ventral part of the AVG in j′ and k′, respectively, where BrdU incorporation is very low. Representative images of six otic vesicles per condition and from at least three independent experiments are shown, and they were obtained from compiled confocal microscopy projections of otic vesicles. Orientation, A, anterior; D, dorsal. Scale bars, 150 µm. (**B**) Quantification (described in Materials and Methods) of the areas (µm^2^) of both otic vesicle (circles) and AVG (triangles) in the presence or absence of IGF-I (closed and open figures, respectively). Data are shown as mean±SEM. Statistical significance between the control and IGF-I conditions at each time-point studied was estimated by ANOVA, followed by Bonferroni and Tukey's multiple comparisons: *P<0.05, ***P<0.001 (0S versus IGF-I at each time-point).

To study the state of proliferation in the IGF-I-cultures, otic vesicles were cultured for different periods of time (1, 4, 8, 20 or 30 h) in the presence of BrdU, in both 0S and IGF-I conditions (Figure 3 A). During the first 4 h, BrdU uptake by otic vesicles was similar in the IGF-I and 0S conditions (Figure 3 A, compare a′ with g′, and b′with h′). After 8 h in culture, BrdU incorporation in the 0S condition was observed in the AVG and in the ventromedial aspect of the otic vesicle (Figure 3 A, c′, arrow and arrowhead, respectively; Figure S2 A). BrdU incorporation was negligible after 20 and 30 h (Figure 3 A, d′ and e′). Interestingly, after 20 h in culture in presence of IGF-I, BrdU positive cells were abundant in the AVG, except in its most ventral aspect (Figure 3 A, j′, arrow; detail in j″). This trait was more evident after 30 h in culture, where negligible IGF-I-induced BrdU incorporation was observed in the ventral aspect of the AVG (Figure 3 A, k′, arrow; detail in k″). The areas of both the otic vesicle and the AVG were measured in both 0S and IGF-I conditions for the different time-points (Figure 3 B). There were no significant differences in the size of the otic vesicles maintained in the presence or absence of IGF-I up to 4 h in culture. By contrast, from 8 h onwards, the otic vesicles cultured in the presence of IGF-I were significantly larger than those of the 0S condition. The AVG followed a similar trend showing a small but significant increase in size after 8 h in the presence of IGF-I (Figure 3 B). These data indicate that IGF-I promotes a widespread and sustained entry in to S-phase of the cell cycle in cultured otic vesicles.

### Spatiotemporal pattern of cell death in otic vesicle explant cultures

**Figure 4:**
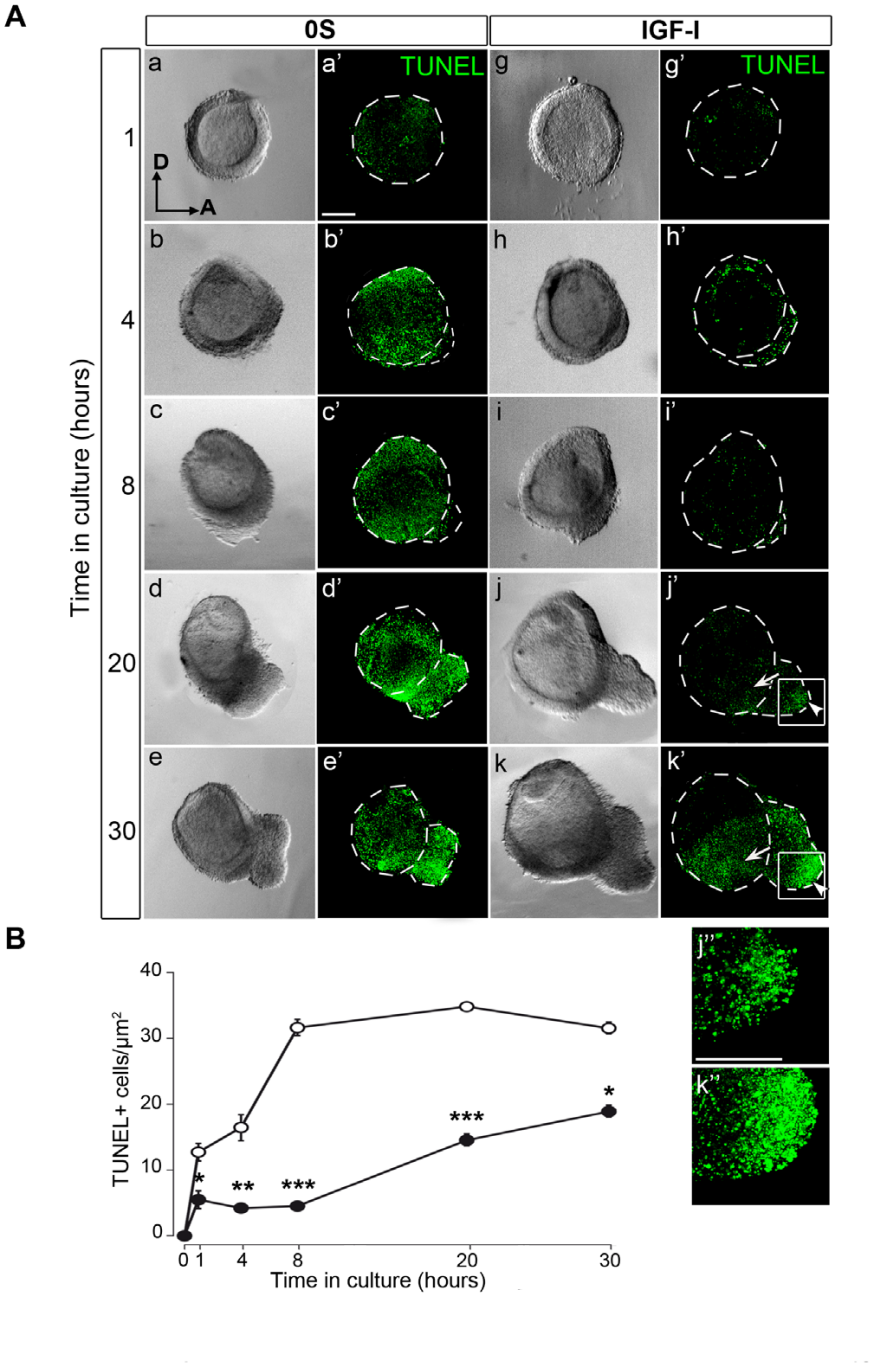
IGF-I protects otic progenitors from programmed cell death. (**A**) Spatiotemporal pattern of TUNEL-positive cells in otic vesicles. Otic vesicles were isolated from HH18 chicken embryos and cultured in serum-free medium either without additives (0S; a–e and a′–e′) or supplemented with IGF-I (10 nM; g–k and g′–k′). Cell death was visualized by TUNEL staining (green, a′–k′). Light microscopy images are also shown for comparison (a–k). Arrows and arrowheads show the accumulation of TUNEL-positive cells in the otic epithelium and in the AVG, respectively (j′, k′). Panels j″ and k″ show the boxed areas of the ventral part of the AVG in j′ and k′ respectively, where TUNEL-positive cells accumulate. Representative images of six otic vesicles per condition and from at least three independent experiments are shown, and they were obtained from compiled confocal microscopy projections of otic vesicles. Orientation: A, anterior; D, dorsal. Scale bars, 150 µm. (**B**) TUNEL-positive cells were counted in the presence and absence of IGF-I (close and open circles, respectively). Data are shown as mean±SEM. Statistical significance between the control and IGF-I conditions at each time-point was estimated by ANOVA and using the Bonferroni and Tukey's post-hoc tests for multiple comparisons: *P<0.05, **P< 0.01 and ***P<0.001 (0S versus IGF-I at each time-point).

To study the survival of otic neural progenitors, we performed TUNEL labelling in otic vesicles, cultured for different periods of time (1, 4, 8, 20 or 30 h), in both 0S and IGF-I conditions (Figure 4 A). TUNEL-positive cells were counted at the different time-points (Figure 4 B). Otic vesicles cultured in control 0S conditions showed apoptosis that could be rescued in the presence of either IGF-I or FBS to the culture medium (Figure S2 B). In the 0S otic vesicles, TUNEL staining increased rapidly during the first 8 h in culture, and afterwards it remained elevated and widespread (Figure 4 A, a′–e′). Although in the presence of IGF-I there was also a time-dependent increase in TUNEL staining, it was significantly reduced compared with the 0S condition (Figure 4 A, g′–k′). In addition, the distribution of TUNEL-positive cells was different. In the IGF-I-treated explants, up to 20 h of culture, TUNEL-positive cells were restricted to the ventromedial neurogenic zone of the otic vesicle (Figure 4 A, j′ and k′, arrows) and to the ventral part of the AVG (Figure 4 A, arrowheads in j′ and k′; details in j″ and k″). Accordingly, this area showed negligible BrdU incorporation even in the presence of IGF-I (see Figure 3 A, j′, k′, j″ and k″). These data indicate that IGF-I protects otic progenitors in culture from apoptosis and that as development proceeds the most mature neural cells present high TUNEL-staining, even in the presence of IGF-I.

### IGF-I protects otic progenitors from caspase-3-dependent programmed cell death

**Figure 5:**
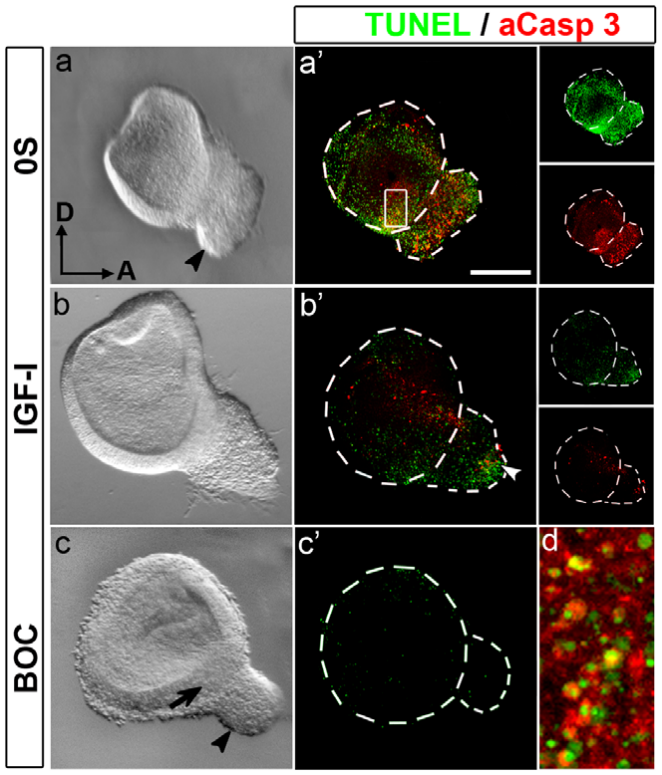
IGF-I protects otic progenitors from caspase-3-dependent apoptosis. Otic vesicles were isolated from HH18 chicken embryos and cultured for 20 h in serum-free culture medium without additives (0S; a and a′), in the presence of IGF-I (10 nM; b and b′) or the pan-caspase inhibitor BOC (50 µM; c and c′). Cell death was visualized by TUNEL staining (green), and immunostaining for activated-caspase-3 (red) was performed. Light microscopy images are also shown for comparison (a–c). BOC treatment caused a reduction in the AVG size (c, arrowhead) and the otic epithelium appeared thickened (c, arrow). The boxed area in a′ is shown at a higher magnification in d, which shows the TUNEL-positive nuclei surrounded by activated caspase-3. Representative images of six otic vesicles per condition and from at least three independent experiments are shown, and they were obtained from compiled confocal microscopy projections of otic vesicles. A, anterior; D, dorsal. Scale bar, 150 µm.

To determine whether IGF-I-abolished programmed cell death was caspase-dependent, we studied active caspase-3 expression levels (Figure 5). Explants in the 0S condition, showed areas of intense apoptotic cell death where TUNEL-labelled apoptotic nuclei were surrounded by cytoplasm containing active caspase-3 (Figure 5, a′; detail in d). The addition of exogenous IGF-I caused a striking reduction in both TUNEL and active caspase-3 labelling, although some stained regions could still be seen, in accord with previously shown data (Figure 5, b′; see Figure 4). IGF-I also promoted otic epithelium morphogenesis (Figure 5, compare a with b). In the absence of IGF-I, treatment with Boc-D-FMK, a cell-permeable irreversible inhibitor of caspases, blocked cell death (Figure 5, c′), increased otic vesicle size, and interestingly, caused an abnormal thickening in the otic epithelium that showed a rounded morphology, and also caused a reduction in the AVG size (Figure 5, compare a with c, arrow and arrowheads, respectively). These data suggest that apoptosis is required for morphogenesis and neurogenesis.

### Activation of the PI3K-AKT pathway is required for IGF-I-mediated survival of otic neuroblasts

**Figure 6:**
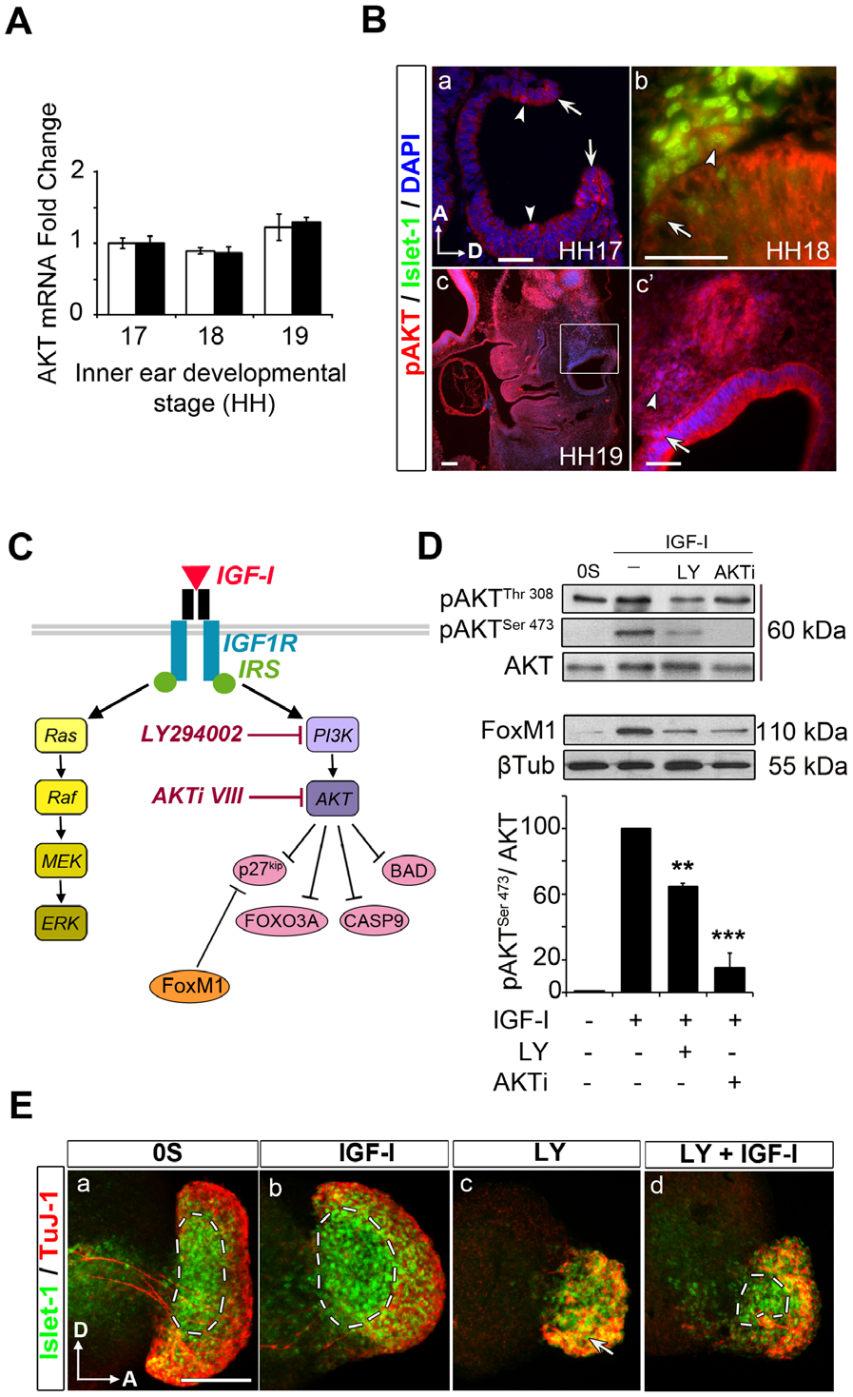
The PI3K-AKT pathway mediates IGF-I survival of otic neuroblasts. (**A**) Expression of inner ear *Akt1* (white bars) and *Akt3* (black bars) mRNA analyzed by qRT-PCR at different stages, using eukaryotic 18s rRNA as the endogenous control gene. Gene expression was calculated as 2 ^−ΔΔCt^ and normalized to the levels at HH17. The results are expressed as the means±SEM from four independent experiments performed in triplicate. (**B**) Distribution of activated AKT (red, pAKT, Ser 473) in the developing inner ear. At HH17, pAKT is strongly expressed at the edges of the otic pore and in the mitotic epithelial cells (a, arrows and arrowheads respectively). At stage HH18, both epithelial and migrating Islet-1-positive otic neuroblasts (green) accumulate cytoplasmic pAKT (b, arrow and arrowhead, respectively). At stage HH19, the otic epithelium and ganglionic neuroblasts strongly express pAKT (c and c′, arrow and arrowheads, respectively). Panel c′ corresponds to the boxed area in c. Orientation: A, anterior; D, dorsal. (**C**) Schematic representation of the two main signaling cascades triggered by IGF-I: PI3K-AKT and RAF-MEK-ERK. LY294002 (LY) inhibits PI3K activation, whilst AKTi VIII (AKTi) inhibits AKT phosphorylation. The arrows denote the facilitating action whereas crosses indicate inhibitory influence. (**D**) Levels of activated AKT (pAKT), AKT and FoxM1 with different treatments. Both pAKT and FoxM1 levels are increased in presence of IGF-I. Quiescent HH18 otic vesicles (15 per condition) were incubated for 30 min (pAKT Ser473 and Thr 308) or 20 h (FoxM1) in the presence of IGF-I (10 nM), LY (25 µM), AKTi, (50 µM), or combinations of IGF-I and the inhibitors. Representative blots are shown from four independent experiments. The data are shown as the mean±SEM and the statistical significance between the different conditions was estimated by ANOVA: **P<0.01, ***P<0.005, versus IGF-I. (**E**) Effects of AKT-inhibition on Islet-1-positive neuroblast population. Otic vesicles were isolated from HH18 chicken embryos and cultured for 20 h in 0S culture conditions (a), in the presence of IGF-I (10 nM, b), LY (25 µM, c), or a combination of both (d). Immunostaining for TuJ-1 (red) and Islet-1 (green) were performed. Islet-1-positive population increases in the presence of IGF-I compared to 0S (a and b, dashed areas). In the presence of LY, there are fewer Islet-1-positive neuroblasts and generalized TuJ-1 expression in the AVG (c, arrow). In the presence of LY and IGF-I, the Islet-1-positive population partially recovers (d, dashed areas). Representative images of five otic vesicles per condition and from at least three independent experiments are shown, and they were obtained from compiled confocal microscopy projections of otic vesicles. Orientation: A, anterior; D, dorsal. Scale bar, 150 µm.

*Akt* gene expression was studied in the inner ear at selected stages of otic development by quantitative RT-PCR. Transcripts encoding for *Akt1* and *Akt3* were expressed in comparable levels from HH17 to HH19 (Figure 6 A). AKT activation by phosphorylation (pAKT), was analysed in chicken embryos at the stages HH17 to HH19 (Figure 6 B). At HH17, pAKT was detected strongly at the closing edges of the otic pore and in mitotic cells of the otic epithelium (Figure 6 B, a, arrows and arrowheads, respectively). At stage HH18, pAKT was detected in the epithelial and the migrating neuroblasts (Figure 6 B, b arrow and arrowhead respectively). At HH19, pAKT was shown in the otic epithelium, and it was abundant in the neurogenic region and in the AVG (Figure 6 B, c and c′, arrow and arrowhead, respectively). The effects of IGF-I on survival and apoptosis involve signalling through the PI3K-AKT pathway in most cell contexts. Specific drugs such as the PI3K inhibitor LY294002 (LY) and the AKT inhibitor AKTi VIII (AKTi) can be used to study the consequences of PI3K-AKT pathway impairment (Figure 6 C). Upon the addition of IGF-I to otic vesicles in culture, the levels of pAKT increased, and this effect was abolished by pre-treatment with both inhibitors LY and AKTi (Figure 6 D). Neither inhibitor caused significant changes in the pERK/ERK ratio determined in parallel experiments (data not shown). Interestingly, basal phosphorylation of AKT was detected at threonine 308 (Thr 308) but not at the serine 473 (Ser 473) residue, and while AKTi had no effect on the former, LY reduced both phosphorylations (Figure 6 D). Overnight AKT inhibition also triggered a decrease in FoxM1 levels, a transcriptional activator of the G2-M-specific gene cluster that promotes p27^Kip^ degradation [45] and is modulated by IGF-I in the mouse cochlea [7] (Figure 6 D).

To study the effects of AKT inhibition in cultured otic vesicles, explants were cultured for 20 h in the presence or absence of IGF-I, LY or both. In accord with previously shown data, IGF-I treatment caused an increase in the Islet-1-positive and TuJ-1-negative neuroblast population, when compared to the 0S condition (Figure 6 E, compare a and b dashed areas). LY treatment caused a decrease in the Islet-1-positive neuroblasts, whereas mature TuJ-1-positive neuroblasts survived, although they did not show neurite extension (Figure 6 E, c, arrow). Exposure to IGF-I partially recovered the neuroblast population after LY treatment (Figure 6 E, d, dashed area). IGF-I-treated otic vesicles showed a wide expression of pAKT that was abundant in ganglionic neuroblasts. Exposure to LY dramatically decreased the pAKT levels in both the otic epithelium and the AVG (data not shown).

### Epithelial and ganglionic neuroblast populations are reduced upon AKT-inhibition

**Figure 7:**
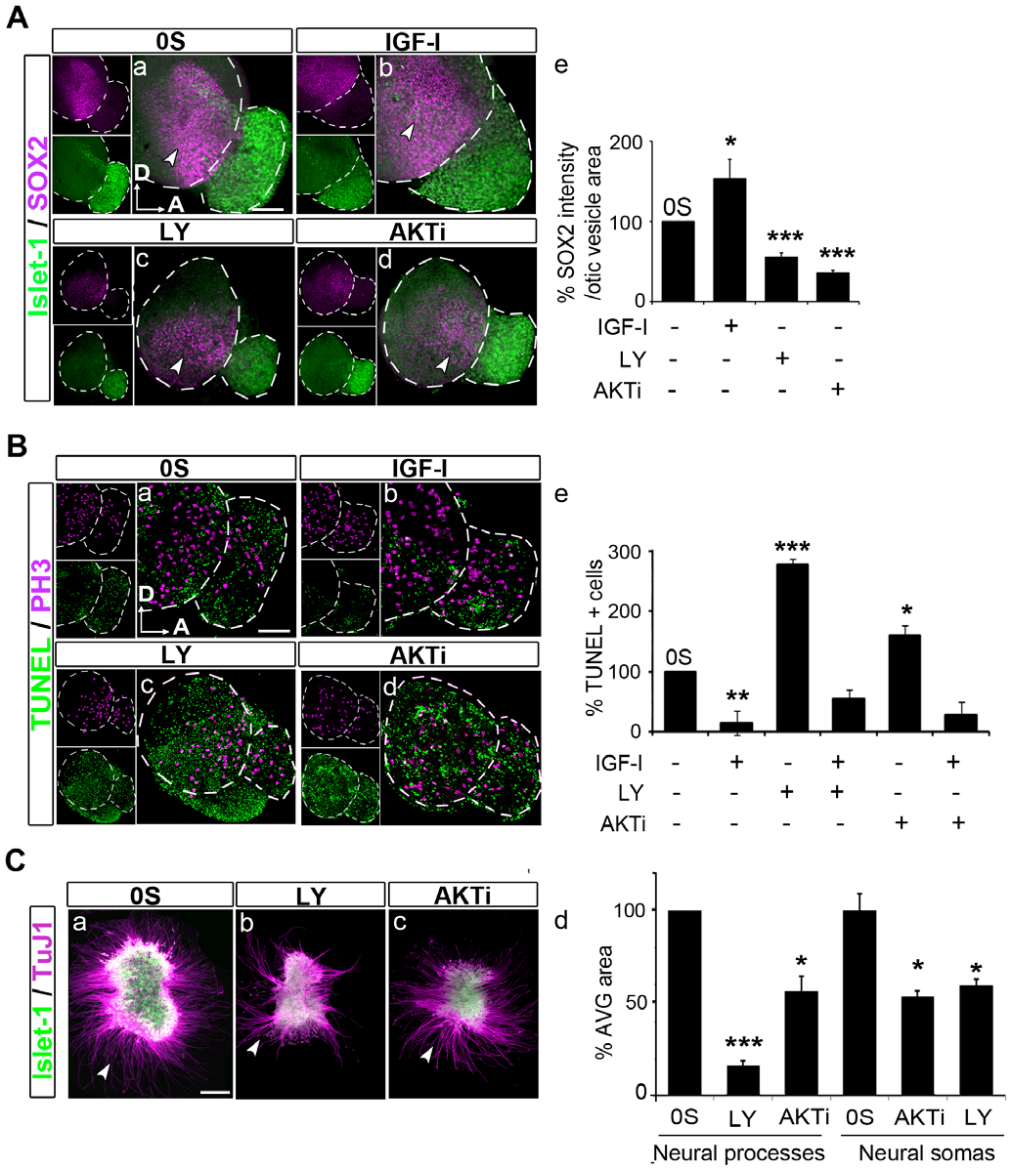
AKT inhibition decreases neuroblast populations. (**A**) SOX2 expression upon PI3K-AKT inhibition. Otic vesicles were isolated from HH18 chicken embryos, made quiescent and cultured for 20 h in serum-free culture medium without additions (0S, a) or in the presence of IGF-I (10 nM, b), LY294002 (LY, 25 µM, c) or AKTi VIII (AKTi, 50 µM, d). Double immunostaining was carried out for SOX2 (magenta) and for the neuroblast nuclear marker Islet-1 (green). The quantification of SOX2-positive population was performed as described in Materials and Methods). SOX2 is expressed in the otic epithelial neuroblast population (a, arrowhead) and it is significantly increased in presence of IGF-I (b, arrowhead, e) but it is markedly reduced in the presence of either AKTi or LY (c and d, arrowheads, e). Representative images of compiled confocal microscopy projections are shown from at least six otic vesicles per condition from at least three independent experiments. All otic vesicles are orientated in the same way: A, anterior; D, dorsal. Scale bar, 75 µm. The data are shown as the mean ± SEM and the statistical significance between the different conditions was estimated by Student's t-test: *P<0.05, ***P<0.001, versus control (0S). (**B**) TUNEL labeling and PH3 expression upon PI3K-AKT inhibition. Otic vesicles were isolated from HH18 chicken embryos and they were cultured in the same conditions as in A. Apoptotic cell death was visualized by TUNEL (green) in cultured otic vesicles, and immunostaining for the mitotic marker phospho-Histone-3 was performed (PH3, magenta). Both TUNEL and PH3-positive cells were quantified (as described in Materials and Methods). PH3 levels did not vary with the different treatments, but TUNEL levels decreased markedly in the presence of IGF-I and increased dramatically with either inhibitor. Treatment with IGF-I restored low TUNEL-levels (e). The data are shown as the mean±SEM and the statistical significance between the different conditions was estimated by Student's t-test: *P<0.05, **P<0.01 and ***P<0.001 versus control (0S). (**C**) Acoustic-vestibular ganglia (AVG) explants were obtained from HH19 embryos and cultured in serum-free medium for 20 h with no additives (0S, a) or in the presence of LY294002 (LY, 25 µM, b) or AKTi VIII (AKTi, 50 µM, c). Immunostaining for TuJ-1 (magenta) and Islet-1 (green) were performed. Compared to 0S (a, arrowhead), the neuronal soma area of the LY and the AKTi-treated AVG is smaller (d). Areas were measured as described in Materials and Methods (d). The data are shown as the mean±SEM and the statistical significance between the different conditions was estimated by Student's t-test: *P<0.05, **P<0.01, versus control (0S). Scale bars, 75 µm. Representative images from compiled confocal microscopy projections of AVG are shown, from at least six otic vesicles or AVG explants per condition studied in at least three independent experiments.

In order to further study the consequences of PI3K-AKT inhibition in otic neuroblast populations, we explored SOX2 expression (Figure 7 A). The transcription factor SOX2 is essential for the sustained self-renewal of undifferentiated embryonic cells and it is expressed in the otic proneural domain, which contains the epithelial neuroblasts [46], [47]. Whilst the SOX2-positive population was significantly increased by IGF-I treatment (Figure 7 A, compare a with b, arrowheads; quantification in e), there was a dramatic reduction in this population in the presence of either inhibitor (Figure 7 A, compare a with c and d). TUNEL staining and immunodetection of phospho-histone-3 (PH3) were performed to study cell death and cell proliferation respectively upon PI3K-AKT-inhibition (Figure 7 B, a–d). IGF-I increased the number of PH3-positive cells mitotic cells, whereas treatment with LY or AKTi did not significantly alter it (data not shown). However, PI3K-AKT inhibition triggered a widespread TUNEL staining in the otic epithelium and AVG, whilst IGF-I reduced cell death (Figure 7 B, compare a with b and c and d; quantification in e). Moreover, IGF-I restored low levels of TUNEL staining even after pre-treatment with either of the inhibitors (Figure 7 B, quantification in e). The increase in TUNEL-positive cells was more pronounced upon PI3K inhibition (Figure 7 B, c, quantification in e). Both inhibitors affected otic vesicle size, LY-treated and AKTi-treated otic vesicle sizes were 63% and 73% of those of controls, respectively. Whereas the reduction observed in the AVG size were of 55% and 65% after treatment with LY and AKTi, respectively, with respect to 0S otic vesicles (data not shown).

To further explore AKT actions in neural cells, AVG were explanted and cultured for 20 h in the presence of either LY or AKTi. Upon treatments, a reduction in Islet-1-positive neuroblast population was observed (Figure 7 C, compare a with b and c). The Islet-1-positive area of the AVG (neural somas area) was significantly reduced with either inhibitor (Figure 7 C, compare a with b and c; quantification in d). Neurite extension (neural processes area) was also significantly affected upon PI3K and AKT inhibition (Figure 7 C, compare a with b and c, arrowheads; quantification in d).

## Discussion

The mechanisms that control the survival and proliferation of otic neural progenitors are still poorly understood. We show here that otic neural progenitors are protected from programmed cell death by IGF-I, which increases FoxM1 levels and p27Kip-negative neural population, thus maintaining neuroblasts in a proliferative and undifferentiated state. IGF-I promotes survival through the activation of IGF1R, leading to PI3K and AKT activation. Inhibition of AKT causes generalized apoptosis of neural progenitors. Post-mitotic p27^Kip^-positive neurons become independent of IGF-I for survival and, concomitantly, neurotrophin receptors are up-regulated and otic neurons extend the fibers that connect them to the sensory epithelia.

### Otic neuroblasts depend on IGF-I for survival and expansion

Programmed cell death is part of the normal development of the nervous system [8], and it plays an important role during the earliest morphogenetic events of inner ear development [5], [6]. Apoptosis contributes to the regulation of cell number in the epithelial and ganglionar neuroblast populations, and it serves to remove aberrant cells. Total blockage of apoptosis with the caspase inhibitor Boc-D-FMK causes malformations in the otic vesicle, including the thickening of the neurogenic area in the otic epithelium, which suggests an impairment on neural progenitor migration from the otic epithelium. Accordingly, the size of the associated AVG appears reduced. This suggests that programmed cell death fulfils a complementary role in the epithelial neurogenic area, allowing the neuroblasts to detach from the epithelium. IGF-I reduces caspase-3 activation and TUNEL staining. However, the associated cell survival is spatiotemporally regulated so that it results in the persistence of specific spots of cell death. After IGF-I addition, apoptosis was negligible during the first 8 h in culture, suggesting that it protects undifferentiated otic progenitors. Apoptosis always increases after long periods in culture (24 h), and, even in presence of IGF-I, there is a localized region of cell death that corresponds to G4/p27^Kip^ positive post-mitotic neurons. Another region of cell death can be observed in the otic epithelium and corresponds with the neurogenic region. This area of cell death is also observed *in vivo* [6]. Otic neurons depend on brain-derived neurotrophic factor and/or neurotrophin-3, which are provided by the peripheral sensory system. It is likely that the absence of such neurotrophins causes neurotrophic cell death, a common mechanism to adjust neuronal numbers and to eliminate unhealthy or undesired cells [48].

Our results show that in the developing inner ear, IGF-I-dependent neural survival is marked by the state of differentiation and the microenvironment, and it occurs within a time-window that precedes neurotrophin-dependence. IGF-I protects otic precursors so they continue to proliferate for longer periods. Proliferation in combination with a decreased rate of cell death results in an increase in the size of the otic vesicle. IGF-I increased the population of neuroblasts (Islet-1-positive), while reducing that of mature neurons (G4- and p27^Kip^-positive) which become confined to the ventral aspect of the AVG. Accordingly, cell death occurs in post-mitotic neurons that down-regulate the expression of high affinity IGF-I receptors.

### IGF-I acts through the PI3K-AKT pathway to promote the survival of neural progenitors

Here we show that the survival actions of IGF-I on otic neuroblasts are mediated by the activation of AKT via PI3K. AKT expression is regulated during early inner ear development. Active pAKT is present in the otic epithelium and in migrating and ganglion neuroblasts, as well as in otic neurons. Blockade of either PI3K or AKT activity causes a dramatic loss of both epithelial and ganglionic neuroblast populations. IGF-I partially rescues this phenotype possibly by activating alternate pathways, including those initiated by CRAF activation, which has been shown to promote survival in the otic vesicle [20]. It is worth noting that blocking PI3K causes both an increase in apoptosis and other morphological alterations which are not observed when AKT activity is inhibited. Members of the PI3K family regulate apoptosis but they also participate in other biological processes including autophagy [49], [50]. AKT inhibition increases apoptosis and decreases the number of SOX2-positive epithelial precursors and of Islet-1-positive/TuJ-1-negative immature neuroblasts. SOX2 has been proposed as a marker of otic progenitors committed to a neural fate [46]. AKT does not affect the mature ganglionic population, but neurons did not extend neural processes, suggesting a role for AKT in differentiation. Indeed, AKT has been implicated in neurite outgrowth in other systems [51]. AKT has also been reported to directly modulate cell cycle, thus AKT phosphorylates and promotes the accumulation in the cytoplasm of Skp2, resulting in p27 ubiquitylation, degradation and cell cycle activation [28], [27].

There is previous evidence that IGF-I can promote neurogenesis [52], proliferation of neural progenitors [53], [54], [55], and proliferation of neural stem cells in culture [56]. Depending on the cell type and context, these actions of IGF-I are mediated by distinct signaling pathways [16], [20], [57], [58]. The basic mechanism underlying these actions is the capacity of IGF-I to promote G_1_/S cell cycle progression by regulating cyclin kinase activation via the activation of specific signaling pathways [55], [59]. IGF-I regulates the nuclear levels of cyclin-dependent kinase inhibitor p27^Kip^ in the rat cerebral cortex [55], and in the developing mouse cochlea [21]. FoxM1 transcription factor is an intermediate step in the inhibition of cell-cycle inhibitors p21^Cip^ and p27^Kip^ [60], [61]. Indeed, *FoxM1* knock-out mice are embryonic lethal due to the lack of progenitor cell proliferation [62]. Here, we show that IGF-I acts via AKT/FoxM1 to promote long-term maintenance of neural otic precursors with the concomitant decrease of p27^Kip^-positive cell numbers. As the expression of IGF-I high affinity receptors is reduced, otic neurons mature and express cyclin inhibitor p27^Kip^.

In summary, we show here that the IGF-I/AKT signalling pathway is fundamental for survival of proliferative otic neuroblasts and the maintenance of the undifferentiated state. This suggests a crucial role of this pathway in establishing the final number of neurons and the timing at which neuron generation proceeds during otic development.

## Supporting Information

Figure S1 **Glial SOX10 expression in the inner ear.** SOX10 (red) and Islet-1 (green) expression was studied by immunohistochemistry in HH19 chicken embryo sections (a, b) and in cultured otic vesicles (c, d). SOX10 was labelling neural crest glial cells within the AVG (a, b, arrowheads). In cultured OV there were no detectable SOX10-positive cells associated to the neuroblasts that express Islet-1 (c, d). Panels b and d correspond to the boxed areas in a and c, respectively. Representative microphotographs are shown from at least two embryos and six otic vesicles. Orientation: A, anterior; D, dorsal. Scale bars, 150 µm.
(TIF)

Figure S2 **IGF-I promotes proliferation and survival in otic neuroblasts.** (**A**) IGF-I promotes BrdU incorporation in epithelial neuroblasts. Otic vesicles were isolated from HH18 chicken embryos and cultured in serum-free medium without additives (0S; s) or supplemented with IGF-I (10 nM; b). Immunostaining for incorporated BrdU was performed (green). Representative images from confocal optic planes of BrdU incorporation in cultured otic vesicles are shown. The arrowheads point to the accumulation of BrdU-positive cells in the neurogenic region in the otic epithelium from the IGF-I condition. (**B**) IGF-I protects otic progenitors from programmed cell death induced by serum deprivation. Otic vesicles were isolated from HH18 chicken embryos and cultured for 4 (a–c) or 20 hours (d–f) in serum-free medium either without additives (0S; a, d) or supplemented either with IGF-I (10 nM; b, e) or FBS (2.5% v/v c, f). Cell death was visualized by TUNEL staining (green). Representative images of six otic vesicles per condition and from at least three independent experiments are shown, and they were obtained from compiled confocal microscopy projections of otic vesicles. Orientation, A, anterior; D, dorsal. Scale bars, 150 µm.
(TIF)
