# Supplementary figures and images for: AKT Signaling Mediates IGF-I Survival Actions on Otic Neural Progenitors
Source: PLoS One. 2012 Jan 23;7(1):e30790. doi: 10.1371/journal.pone.0030790 (PMC3264639; doi:10.1371/journal.pone.0030790)

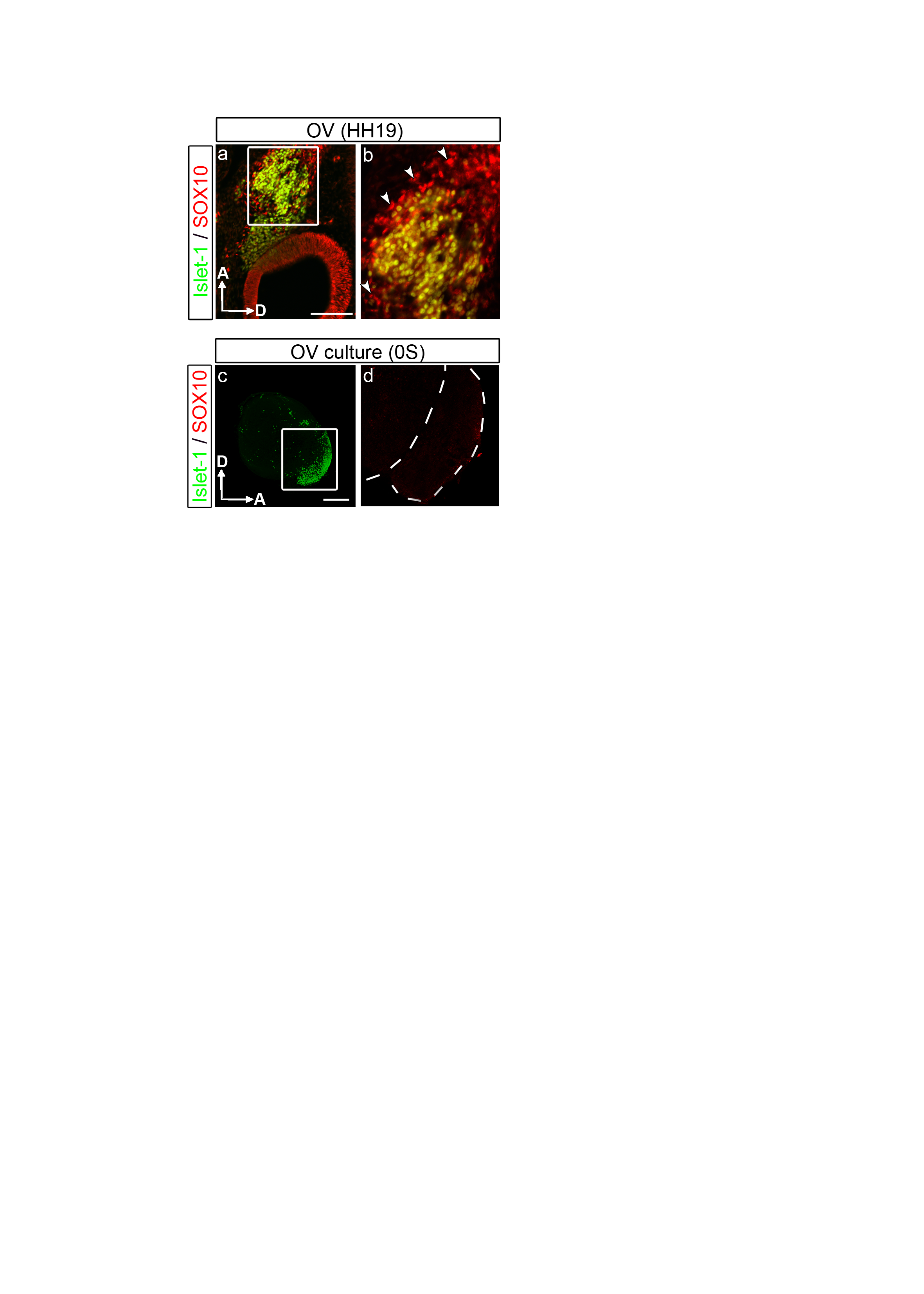

Supplement: Figure S1 — Glial SOX10 expression in the inner ear. SOX10 (red) and Islet-1 (green) expression was studied by immunohistochemistry in HH19 chicken embryo sections (a, b) and in cultured otic vesicles (c, d). SOX10 was labelling neural crest glial cells within the AVG (a, b, arrowheads). In cultured OV there were no detectable SOX10-positive cells associated to the neuroblasts that express Islet-1 (c, d). Panels b and d correspond to the boxed areas in a and c, respectively. Representative microphotographs are shown from at least two embryos and six otic vesicles. Orientation: A, anterior; D, dorsal. Scale bars, 150 µm. (TIF) [file pone.0030790.s001.tif]

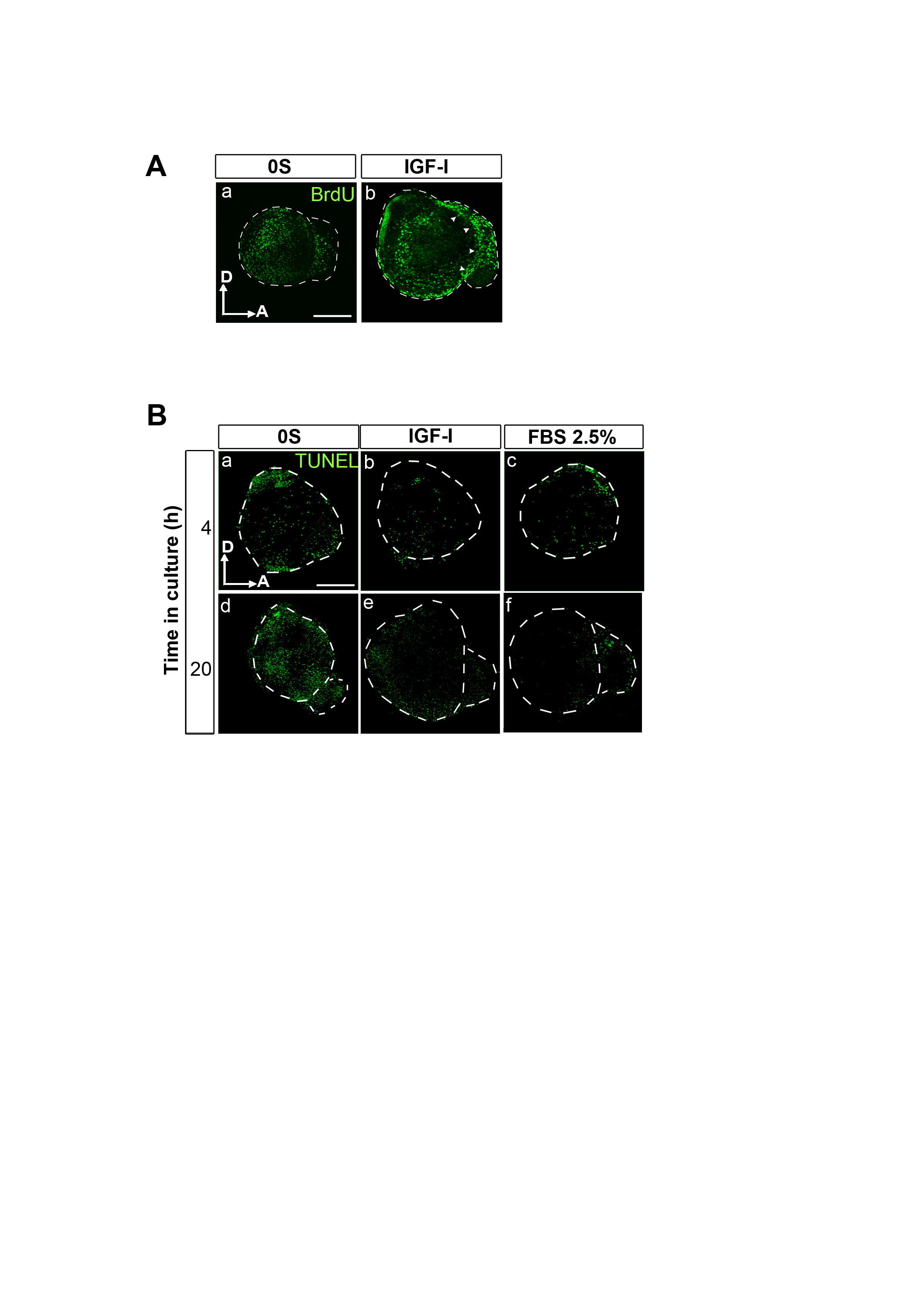

Supplement: Figure S2 — IGF-I promotes proliferation and survival in otic neuroblasts. (A) IGF-I promotes BrdU incorporation in epithelial neuroblasts. Otic vesicles were isolated from HH18 chicken embryos and cultured in serum-free medium without additives (0S; s) or supplemented with IGF-I (10 nM; b). Immunostaining for incorporated BrdU was performed (green). Representative images from confocal optic planes of BrdU incorporation in cultured otic vesicles are shown. The arrowheads point to the accumulation of BrdU-positive cells in the neurogenic region in the otic epithelium from the IGF-I condition. (B) IGF-I protects otic progenitors from programmed cell death induced by serum deprivation. Otic vesicles were isolated from HH18 chicken embryos and cultured for 4 (a–c) or 20 hours (d–f) in serum-free medium either without additives (0S; a, d) or supplemented either with IGF-I (10 nM; b, e) or FBS (2.5% v/v c, f). Cell death was visualized by TUNEL staining (green). Representative images of six otic vesicles per condition and from at least three independent experiments are shown, and they were obtained from compiled confocal microscopy projections of otic vesicles. Orientation, A, anterior; D, dorsal. Scale bars, 150 µm. (TIF) [file pone.0030790.s002.tif]
